# Evidence for the agricultural origin of resistance to multiple antimicrobials in *Aspergillus fumigatus*, a fungal pathogen of humans

**DOI:** 10.1093/g3journal/jkab427

**Published:** 2021-12-13

**Authors:** S Earl Kang, Leilani G Sumabat, Tina Melie, Brandon Mangum, Michelle Momany, Marin T Brewer

**Affiliations:** Fungal Biology Group and Plant Biology Department, University of Georgia, Athens, GA 30602, USA; Fungal Biology Group and Plant Pathology Department, University of Georgia, Athens, GA 30602, USA; Fungal Biology Group and Plant Pathology Department, University of Georgia, Athens, GA 30602, USA; Fungal Biology Group and Plant Biology Department, University of Georgia, Athens, GA 30602, USA; Fungal Biology Group and Plant Pathology Department, University of Georgia, Athens, GA 30602, USA; Fungal Biology Group and Plant Biology Department, University of Georgia, Athens, GA 30602, USA; Fungal Biology Group and Plant Pathology Department, University of Georgia, Athens, GA 30602, USA

**Keywords:** antimicrobial resistance, *Aspergillosis*, azoles, fungicide, *Aspergillus fumigatus*

## Abstract

Pathogen resistance to clinical antimicrobial agents is an urgent problem. The fungus *Aspergillus fumigatus* causes 300,000 life-threatening infections in susceptible humans annually. Azoles, which are widely used in both clinical and agricultural settings, are currently the most effective treatment, but resistance to clinical azoles is emerging worldwide. Here, we report the isolation and analysis of azole-sensitive and azole-resistant *A. fumigatus* from agricultural environments in the southeastern United States (USA) and show that the USA pan-azole-resistant isolates form a clade with pan-azole-resistant isolates from the United Kingdom, the Netherlands, and India. We show that several pan-azole-resistant isolates from agricultural settings in the USA and India also carry alleles with mutations conferring resistance to agricultural fungicides from the benzimidazole (MBC) and quinone outside inhibitor (QoI) classes. We further show that pan-azole-resistant *A. fumigatus* isolates from patients in clinical settings in the USA, India, and the Netherlands also carry alleles conferring resistance to MBC and QoI agricultural fungicides. The presence of markers for resistance to agricultural-use fungicides in clinical *A. fumigatus* isolates is strong evidence for an agricultural origin of pan-azole resistance in patients. The presence of multiple fungicide-resistance alleles in agricultural and clinical isolates further suggests that the unique genetics of the pan-azole-resistant clade enables the evolution and/or persistence of antimicrobial resistance mutations leading to the establishment of multifungicide-resistant isolates.

## Introduction

Fungi are important pathogens of humans, causing over 1.5 million deaths annually (Brown *et al.* 2012). Fungi are also important pathogens of plants, causing crop losses of 20%, and postharvest losses of 10% (Fisher *et al.* 2018). Azole antimicrobials target ergosterol synthesis and are highly effective against fungal pathogens of both humans and plants leading to their widespread use in clinical and agricultural settings (Sheehan *et al.* 1999; Klittich 2008). The filamentous fungus *Aspergillus fumigatus* is a saprobe found in a variety of environments including soil, compost, and decaying plant material; however, in immunocompromised individuals it can cause the devastating disease aspergillosis. It causes 300,000 life-threatening infections in susceptible human hosts annually and azoles are the most effective treatment (Patterson *et al.* 2016). During the last decade Europe and Asia have seen an alarming increase in azole-resistant *A. fumigatus* in the clinic and azole resistance is now present on six continents (Sharpe *et al.* 2018).

Though some resistance has been associated with long-term azole therapy in patients with chronic infections, at least two-thirds of patients with azole-resistant *A. fumigatus* infections have not previously undergone azole therapy (Snelders *et al.* 2009; Bowyer and Denning 2014). Thus, the environmental use of azoles has been proposed to be the driving force for the majority of clinical resistance in *A. fumigatus* with several studies suggesting that most azole-resistant isolates originated from widespread agricultural use of azoles to combat plant-pathogenic fungi (Snelders *et al.* 2009; Chowdhary *et al.* 2013; Berger *et al.* 2017). Moreover, the same mutations in *cyp51A—*which encodes the ergosterol biosynthetic protein targeted by azoles—have been reported in isolates from both clinical and agricultural settings in Europe, Asia, Africa, the Middle East, and North and South America (Snelders *et al.* 2009; Bowyer and Denning 2014; Vazquez and Manavathu 2016; Verweij *et al.* 2016; Wiederhold *et al.* 2016; Alvarez-Moreno *et al.* 2017; Hurst *et al.* 2017; Moore *et al.* 2017; Berkow *et al.* 2018; Sharpe *et al.* 2018; Alvarez-Moreno *et al.* 2019). Several point mutations and tandem repeats (TR) within the promoter, including TR_34_/L98H and TR_46_/Y121F/T289A, are commonly associated with azole resistance in environmental isolates. Isolates with the TR_34_/L98H and TR_46_/Y121F/T289A alleles show high levels of resistance to multiple azole drugs (pan-azole resistance) and patients infected with these isolates have higher rates of treatment failure and death (van der Linden *et al.* 2011). Azole-resistant clones with identical microsatellite genotypes have been shown to be globally distributed in both clinical and environmental settings (Sewell *et al.* 2019). Clonal genotypes and the presence of TR_34_/L98H and TR_46_/Y121F/T289A alleles in both agricultural and clinical isolates suggest that pan-azole-resistant clinical strains of *A. fumigatus* might have had an agricultural origin; however, some still argue that pan-azole resistance could have originated independently in the clinic (Gisi 2014; Hollomon 2017).

We hypothesized that if clinical pan-azole resistance originated in agricultural environments, some *A. fumigatus* isolates from clinical settings should show resistance to agricultural fungicides. To delay the evolution and spread of antifungal resistance in agricultural settings, azoles are generally applied to crops in alternation or combination with other fungicides such as the quinone outside inhibitors (QoIs) and, to a lesser extent, benzimidazoles (MBCs) (FRAC 2010; Hobbelen *et al.* 2013). QoI fungicides are widely used on crops, but not on patients (Fernandez-Ortuno *et al.* 2010). MBC fungicides were widely used on crops in the USA in the 1970s, but are much less common in agriculture now due to resistance development by most plant-pathogenic fungi they were used against. MBCs are still used clinically as antihelminthics (Fernando *et al.* 2016). Both QoI and MBC classes are single-site fungicides that have developed resistance rapidly after introduction. The QoI fungicides target a region of the mitochondrial cytochrome bc1 enzyme complex that is highly conserved. The G143A mutation in cytochrome B (cytB) has been shown to cause QoI resistance in dozens of plant-pathogenic fungal species as well as the budding yeast *Saccharomyces cerevisiae* (Gisi *et al.* 2002; Fisher and Meunier 2005; Fernandez-Ortuno *et al.* 2008; Lucas *et al.* 2015). In fact, the G143A association with QoI resistance is so consistent that it is routinely used to screen fields for QoI-resistant plant-pathogenic fungi (Fontaine *et al.* 2009; Pieczul and Wąsowska 2017; Forcelini *et al.* 2018). Similarly, the MBC fungicide benomyl targets a region of β-tubulin that is highly conserved across kingdoms. The F200Y mutation in benA has been reported to cause resistance to benomyl in multiple fungal species (Hawkins and Fraaije 2016). The F200Y mutation and related mutations have also been analyzed in structure–function studies in model organisms including *S. cerevisiae* and *Aspergillus nidulans*, a close relative of *A. fumigatus* (Hawkins and Fraaije 2016). Therefore, the G143A allele in *cytB* or F200Y allele in *benA* (F219Y in *A. fumigatus*) indicate isolates are resistant to QoI or MBC fungicides, respectively. Isolates of *A. fumigatus* from agricultural environments and air samples in Europe have recently been found to carry these alleles and show resistance to QoI and MBC fungicides (Fraaije *et al.* 2020), but alleles encoding resistance to agricultural fungicides have not been documented in isolates from patients.

Very little information is available about the frequency of azole-resistant *A. fumigatus* in agricultural settings in the USA. Azole-resistant *A. fumigatus* isolates with TR_34_/L98H alleles were detected in a peanut field in Georgia (Hurst *et al.* 2017) and strains with TR_34_/L98H and TR_46_/Y121F/T289A alleles have been recently reported in patients (Vazquez and Manavathu 2016; Wiederhold *et al.* 2016). In this study, we isolated azole-sensitive and azole-resistant *A. fumigatus* from agricultural environments in the southeastern USA and identified alleles associated with resistance to azoles and to MBC and QoI fungicides. Our analysis of three loci associated with antifungal resistance in USA isolates from agricultural settings as well as in worldwide environmental and clinical isolates showed resistance alleles for multiple fungicides in both environmental and clinical azole-resistant *A. fumigatus* and shows strong support for an agricultural origin of at least some clinical pan-azole-resistance. Our analysis also showed that pan-azole-resistant isolates from around the world grouped in a single clade and suggests that the unique genetics of the pan-azole-resistant clade enables the evolution and/or persistence of antimicrobial resistance mutations leading to the establishment of multifungicide-resistant isolates. While this paper was in review another study was published that showed similar results (Gonzalez-Jimenez *et al.* 2021).

## Materials and methods

### Sampling

Between July 2017 and March 2018, we collected soil, plant debris, or compost from 56 agricultural sites in Georgia or Florida, USA, including 53 sites that had recently been treated with triazole fungicides, two sites that were in organic production with no triazole use in at least 10 years, and one compost pile with an unknown history of fungicide use on the plant material prior to composting (Supplementary Table S1). Each site was defined as a different field location, different crop at the same field location, or different triazole fungicide treatment. When soil was sampled, plant debris on the soil surface was included if available. If larger piles of debris were present on the soil surface they were collected separately. Soil was sampled by taking 5–10 soil cores to a depth of approximately 10 cm. Plant debris was sampled from the soil surface, cull piles at farms, and waste piles at pecan processing facilities. For each site we collected four samples at separate locations to minimize the collection of clones. Samples were stored in sealed plastic bags for transport and stored open to allow for gas exchange at 4°C for 2–20 days.

### Isolation and storage

The samples were processed as described previously by Snelders *et al.* (2009) and Hurst *et al.* (2017) with some modifications. Briefly, 2 g of soil was suspended in 15 ml of sterile 0.1 M sodium pyrophosphate. Samples were vortexed for 30 s and allowed to settle for 1 min. From the supernatant, 100 μl was pipetted onto Sabouraud dextrose agar (SDA) in 100-mm Petri dishes supplemented with 50 μg/ml chloramphenicol (Sigma Aldrich) and 5 μg/ml gentamicin (Research Products International). The dishes were incubated for 2–4 days at 45°C. Colonies of *A. fumigatus* were initially identified based on morphology and screened for azole resistance on SDA supplemented with 3 μg/ml tebuconazole (TEB; Bayer Corp). Many of the isolates that grew on 3 μg/ml TEB-amended solid medium were not able to grow at similar concentrations of TEB in liquid medium during the minimum inhibitory concentrations (MICs) testing described below; therefore, these isolates are designated as sensitive to TEB in Supplementary Table S1. Isolates were stored at −80°C in 15% glycerol.

### Antifungal susceptibility testing by MIC

One hundred and seventy-two environmental *A. fumigatus* isolates (Supplementary Table S1), and 48 clinical isolates were tested for antifungal susceptibility under conditions described in the Clinical Laboratory Standard Institute broth microdilution method. Antifungals tested included tebuconazole (TEB; TCI America, OR, USA), itraconazole (ITC; Thermo Sci Acros Organics, NJ, USA), voriconazole (VOR; Thermo Sci Acros Organics), and posaconazole (POS; Apexbio Technology, TX, USA) suspended in DMSO. Briefly, isolates were grown on complete media (Momany *et al.* 1999) slants for 5–7 days at 37°C and harvested with 2 ml of 0.02% Tween-20 solution. Spore suspensions were adjusted to 0.09–0.13 OD at 530 nm using a spectrophotometer. One hundred microliters of 2 × 10^4^ to 5 × 10^4^ conidia/ml were added to 100 ml of RPMI 1640 liquid medium (Thermo Sci Gibco, CA, USA) in microtiter plates with final concentrations of antifungals ranging from 0.015 to 16 μg/ml. Plates were incubated at 37°C for 48 h and MIC break points were determined visually. MIC break point was defined as the lowest concentration at which there was 100% inhibition of growth. Assays were repeated for all resistant isolates and most sensitive isolates. For classification of sensitivity or resistance for ITC and VOR, we used the recommended clinical breakpoints of antifungal resistance for *A. fumigatus* (EUCAST 2018) which are >2 μg/ml; however, EUCAST notes there is uncertainty regarding the cutoff values for POS and some data suggest >1 μg/ml, which we use here, may be more relevant.

### DNA extraction

High molecular weight genomic DNA of *A. fumigatus* isolates was extracted using a modified CTAB protocol as described previously (Pitkin *et al.* 1996). Briefly, approximately 100 mg of mycelium collected from cultures that had been incubated overnight in liquid complete medium were transferred to 2 ml tubes containing approximately 200 µl of 0.5-mm disruption glass beads (RPI, catalog number 9831) and three 3-mm steel beads and lyophilized. Lyophilized cells were pulverized using Geno/Grinder at 1750 rpm for 30 s. Pulverized tissue was incubated in 1 ml of CTAB lysis buffer (100 mM Tris pH 8.0, 10 mM EDTA, 1% CTAB, 1% BME) for 30 min at 65°C. The samples were extracted with chloroform (500 µl) twice and DNA in the upper layer was precipitated in ice cold isopropanol. The precipitated DNA samples were washed with 70% ethanol twice, air dried, and dissolved in 100 µl sterile water. DNA was quantified using NanoDrop One (Thermo Sci, NJ, USA).

### cyp51A sequencing

For all environmental isolates in this study, *cyp51A* was PCR-amplified using previously designed primers (Mellado *et al.* 2001). PCRs were performed with the Q5 Hot Start High-Fidelity 2× Master Mix (New England Biolabs) with 100 ng of genomic DNA, 0.5 μM forward primer 5′-CGGGCTGGAGATACTATGGCT-3′ and 0.5 μM reverse primer 5′-GTATAATACACCTATTCCGATCACACC-3′ in 20 μl reactions. PCRs were performed at 98°C for 2 min followed by 30 cycles of 98°C for 15 s, 62°C for 15 s, and 72°C for 2:30 min, followed by a final extension at 72°C for 5 min. Amplicons were sequenced by the Sanger method (Eurofins genomics, USA) using primers 5′-GCATTCTGAAACACGTGCGTAG-3′, 5′-GTCTCCTCGAAATGGTGCCG-3′, and 5′-CGTTCCAAACTCACGGCTGA-3′. Promoter sequences were aligned to A1163 genomic sequence v43 from Ensembl. Coding sequences were translated to protein sequences and aligned to the Cyp51A protein of *A. fumigatus* A1163 (GenBank accession number EDP50065). Sequence analysis was performed using Geneious v11.1.5 (Biomatters, Auckland, NZ).

### Microsatellite genotyping

Nine microsatellite markers (STR*Af* 2A, 2B, 2C, 3A, 3B, 3C, 4A, 4B, and 4C) previously developed for *A. fumigatus* (de Valk *et al.* 2005) were used to genotype 166 environmental isolates (Supplementary Table S1), the reference isolate Af293, and 48 clinical isolates from Georgia or Florida provided by the Mycotic Diseases Group at CDC (Berkow *et al.* 2018). Multiplex PCR was performed using the Type-it Microsatellite PCR kit (Qiagen) following the manufacturer’s protocol, but with the reaction volume modified to 10 μl. Multiplex reactions contained the following: 5 μl of 2× Type-it Master Mix, 1 μl of 10× primer mix (2 μM of each primer in the multiplex), 1 μl of DNA template, and RNAse-free water. Thermal cycling conditions had an initial denaturation at 95°C for 5 min followed by: 28 cycles of 95°C for 30 s, 57°C for 90 s, and 72°C for 30 s and a final elongation of 60°C for 30 min. Amplification of a product was confirmed by electrophoresis on a 1% (w/v) agarose gel with 1× TBE buffer. PCR products were diluted (1:15) then 1 μl of diluted PCR product was added with 0.1 μl of the internal size standard Genescan-500 Liz (Applied Biosystems) and 9.9 μl of Hi-Di formamide (Applied Biosystems). These were then incubated for 5 min at 95°C and placed immediately on ice. Fragment analysis was performed at the Georgia Genomics and Bioinformatics Core (GGBC) on an Applied Biosystems 730×1 96-capillary DNA analyzer. Microsatellite Plugin in Geneious v.6 (Biomatters) was used to score alleles and loci were distinguished based on expected size range. To examine relationships among all isolates and isolates from different environments, a minimum spanning network was constructed using the Bruvo’s genetic distance model (Bruvo *et al.* 2004) in the Poppr package executed in R (Kamvar *et al.* 2014).

### Library preparation and whole genome sequencing

Genomic DNA was sheared to a mean size of 600 bp using a Covaris LE220 focused ultrasonicator (Covaris Inc., Woburn, MA). DNA fragments were Ampure (Beckman Coulter Inc., Indianapolis, IN) cleaned and used to prepare dual-indexed sequencing libraries using NEBNext Ultra library prep reagents (New England Biolabs Inc., Ipswich, MA) and barcoding indices synthesized in the CDC Biotechnology Core Facility. Libraries were analyzed for size and concentration, pooled, and denatured for loading onto flowcell for cluster generation. Sequencing was performed on an Illumina Hiseq using 300 × 300 cycle paired-end sequencing kit. On completion, sequence reads were filtered for read quality, base called, and demultiplexed using Casava v1.8.4. All environmental *Aspergillus fumigatus* sequence files (eAF) were deposited in the NCBI Biosample database [accession number(s) SAMN19975862–SAMN19975957].

### Single nucleotide polymorphism calling and neighbor-joining tree

Cleaned whole-genome sequence reads for each isolate were *de novo* assembled using SPAdes v3.12.0 (Nurk *et al.* 2013) with option –careful and trained to Af293 reference genome (Nierman *et al.* 2005) using option –trusted-contigs. Corrected fasta files generated from SPAdes were then aligned to Af293 reference genome using Burrows–Wheeler Aligner (BWA) alignment tool (Li *et al.* 2009) and duplicate reads were marked using Picard v2.16.0. Single nucleotide polymorphisms (SNPs) were called with SAMtools mpileup v1.6 (Li *et al.* 2009) with option –I to exclude insertions and deletions then with BCFtools v1.9 with option –c. Bases with phred quality score lower than 40 were filtered using SAMtools seqtk v1.2. SNP data were converted into interleaved phylip format and a neighbor-joining tree was constructed using Seaview v4.7 (Gouy *et al.* 2010). Support for internal branches was determined by 100 bootstrap replicates. The tree was visualized and annotated using iTOL: International Tree of Life v5 (Letunic and Bork 2019).

### Genome mining for agricultural fungicide resistance

Whole-genome sequences (Supplementary Table S3) were searched by tblastn (Altschul *et al.* 1990) for *A. fumigatus cyp51A* (GenBank accession number EDP50065), *benA* (GenBank accession number EDP56324), and *cytB* (GenBank accession number AFE02831). Blast hits were extracted from assemblies using BEDtools v2.26.0. Sequence analysis was performed using Geneious v11.1.5 (Biomatters, Auckland, NZ).

### Fungicide-resistance phenotype assays

Sensitivity assays for QoI were conducted in a 100-mm Petri dishes of SDA that contained 10 µg/ml of azoxystrobin (Sigma Aldrich analytical-grade, diluted from 10 mg/ml stock in acetone) and 100 µg/ml salicylhydroxamic acid (SHAM) (Sigma Aldrich analytical-grade, diluted from 100 mg/ml stock in methanol) (Vega *et al.* 2012). Controls were identical except that they lacked azoxystrobin. Sensitivity assays for MBC fungicides were similar, except they contained only 10 µg/ml benomyl (Sigma Aldrich, diluted from10 mg/ml stock in DMSO) in SDA (Summerbell 1993). Controls lacked benomyl. Three azoxystrobin-amended SDA and three control dishes, as well as 3 benomyl-amended and control dishes were inoculated with 100 µl of a 1 × 10^3^ conidia/ml *A. fumigatus* stock, spread using a sterile glass rod, and incubated at 37°C for 23 h at which point microcolonies were counted by eye. The experiment was performed twice.

## Results

To investigate the prevalence of azole-resistant *A. fumigatus* in agricultural environments in the southeastern USA, we collected soil and plant debris from 56 sites across Georgia and Florida, including 53 sites with a history of azole fungicide use, two organic sites with no fungicide use for at least 10 years, and one compost pile of unknown history (Supplementary Table S1). We recovered 700 isolates of *A. fumigatus* from soil, plant debris, and compost. Isolates were screened for sensitivity to the azole fungicide tebuconazole (TEB) that has widespread use in agriculture. Of the 700 isolates collected, 123 (17.6%) grew on solid medium amended with 3 μg/ml TEB. None of the isolates collected from the two organic sites grew on TEB-amended plates.

MICs for TEB, itraconazole (ITC), voriconazole (VOR), and posaconazole (POS) were determined by broth dilution assays for the 123 isolates that grew on TEB-amended solid medium, and for 49 isolates from the same sites that that did not grow on the amended medium. MIC ranged from 0.5 to >16 μg/ml for TEB, 0.5 to 2 μg/ml for ITC, 0.125 to >16 μg/ml for VOR, and 0.06 to 1 μg/ml for POS. Recommended clinical breakpoints of antifungal resistance for *A. fumigatus* (EUCAST 2018) are >2 μg/ml for ITC and VOR and >0.25 μg/ml for POS; however, EUCAST notes there is uncertainty regarding the cutoff values for POS and some data suggest the cutoff value of >1 μg/ml, which we use here, may be more relevant. Although many of the isolates showed low levels of azole resistance, only 12 of the 123 isolates were highly resistant at clinically relevant levels (Supplementary Tables S1 and S2). The 12 isolates exhibited high levels of resistance to both TEB and VOR with MIC ≥ 16 μg/ml, and decreased sensitivity to ITC and POS (Table 1), showing they are pan-azole resistant. Overall, there was a relatively low frequency of pan-azole resistance among *A. fumigatus* isolates collected from agricultural sites in Georgia and Florida; they were only recovered from a single compost pile and pecan debris at a single site (Supplementary Table S1).

**Table 1:** Azole-susceptibility of environmental *A. fumigatus* isolates from Georgia and Florida, USA with nonsynonymous *cyp51A* mutations

Isolates	*cyp51A* genotype^a^	MIC Ranges (**μ**g/ml)^b^			
		TEB	ITC	VOR	POS
**A1163**	WT	1.0	1.0	0.25	0.25
**eAF221**	TR_46_/Y121F/T289A	>16	1.0-2.0	>16	1.0
**eAF222**	TR_46_/Y121F/T289A	>16	2.0	>16	1.0
**eAF228**	TR_46_/Y121F/T289A	>16	1.0-2.0	>16	1.0
**eAF229**	TR_46_/Y121F/T289A	>16	1.0-2.0	>16	1.0
**eAF230**	TR_46_/Y121F/T289A	>16	1.0-2.0	>16	1.0
**eAF231**	TR_46_/Y121F/T289A	>16	1.0-2.0	>16	1.0
**eAF232**	TR_46_/Y121F/T289A	>16	1.0-2.0	>16	1.0
**eAF233**	TR_46_/Y121F/T289A	>16	1.0-2.0	>16	1.0
**eAF234**	TR_46_/Y121F/T289A	>16	1.0-2.0	>16	1.0
**eAF235**	TR_46_/Y121F/T289A	16	1.0	>16	0.5-1.0
**eAF236**	TR_46_/Y121F/T289A	>16	1.0-2.0	>16	1.0
**eAF513**	TR_46_/Y121F/T289A	>16	1.0	>16	1.0
**eAF010**	I242V	1.0-2.0	1.0	0.5	0.5-1.0
**eAF175**	I242V	2.0	1.0	0.25-0.5	0.5
**eAF263**	I242V	2.0	1.0	0.5	0.5
**eAF265**	I242V	2.0	1.0	0.5	0.5
**eAF406**	I242V	2.0	1.0	0.5	0.25
**eAF500**	I242V	2.0	1.0	0.5	0.5
**eAF589**	I242V	2.0	1.0	0.25-0.5	0.25-0.5
**eAF621**	I242V	2.0	1.0	0.5	0.5
**eAF647**	I242V	2.0	1.0	0.25	0.25
**eAF773**	I242V	2.0	1.0	0.5	0.5
**eAF792**	I242V	1.0-2.0	0.5-1.0	0.25-0.5	0.25-0.5
**eAF016**	Y46F/V172M/T248N/E255D/K427E	1.0-2.0	1.0	0.25-0.5	0.5
**eAF082**	Y46F/V172M/T248N/E255D/K427E	1.0-2.0	1.0	0.25-0.5	0.25-0.5
**eAF128**	Y46F/V172M/T248N/E255D/K427E	2.0	1.0	0.5	0.5
**eAF163**	Y46F/V172M/T248N/E255D/K427E	1.0-2.0	1.0	0.25-0.5	0.5
**eAF609**	Y46F/V172M/T248N/E255D/K427E	2.0-4.0	1.0	0.5-1.0	0.25-0.5

a Cyp51A protein GenBank accession number EDP50065.1 used as reference.

b MICs, Minimal Inhibitory Concentrations; TEB, tebuconazole; ITC, itraconazole; VOR, voriconazole; POS, posaconazole. Each assay was conducted twice. A single value is listed if the result was the same for both assays and a range was listed if the results differed between assays.

To determine whether mutations in *cyp51A* were associated with azole resistance, we sequenced 1286 bp of *cyp51A*, including the promoter and downstream regions, for 123 isolates that grew on TEB-amended medium and for 49 TEB-sensitive isolates from the same sites. The 12 pan-azole-resistant isolates had the TR_46_/Y121F/T289A allele (Table 1) that underlies high levels of resistance to VOR (Berger *et al.* 2017; Moore *et al.* 2017). TEB and VOR have similar molecular structures, which could explain why resistant isolates with the TR_46_/Y121F/T289A allele show higher levels of resistance to both of these azoles. Interestingly, we did not detect the TR_34_/L98H allele that is the most prevalent worldwide in azole-resistant environmental and clinical isolates of *A. fumigatus*. Failure to detect the TR_34_/L98H allele suggests that this set of mutations is less prevalent in the areas we sampled. Eleven of the isolates sequenced had the I242V mutation in Cyp51A and 5 had the Y46F/V172M/T248N/E255D/K427E mutations found in the reference isolate Af293. These 16 isolates with nonsynonymous mutations in *cyp51A*, but without TR in the promoter, had slightly elevated MIC values for TEB, VOR, and POS compared with the sensitive reference isolate A1163 (Table 1). Increased MIC values for isolates with these mutations have been described previously (Howard *et al.* 2009; Chowdhary *et al.* 2014; Pham *et al.* 2014; Rivero-Menendez *et al.* 2016); however, it is not clear if these specific mutations are the cause of increased drug resistance.

To determine the relationship of agricultural isolates from Georgia and Florida to clinical isolates from the same region, we used 9 STR*Af* markers (de Valk *et al.* 2005) to genotype the 168 agricultural isolates that we collected along with 48 clinical isolates collected between 2015 and 2017 by the Centers for Disease Control and Prevention (Berkow *et al.* 2018). None of the clinical isolates were azole resistant. Based on microsatellite data almost every isolate had a unique genotype (Supplementary Figure S1). The combined environmental and clinical *A. fumigatus* population from Georgia and Florida showed no genetic structure, except for the pan-azole-resistant agricultural isolates that had the TR_46_/Y121F/T289A allele. These pan-azole-resistant isolates comprised a single lineage and were isolated from a compost pile and pecan debris from a processing facility (Supplementary Table S1). Our results are consistent with previous studies showing that *A. fumigatus* is panmictic with little population structure either by geographic region or clinical *vs* agricultural setting (Snelders *et al.* 2009; Abdolrasouli *et al.* 2015; Sewell *et al.* 2019).

**Figure 1:**
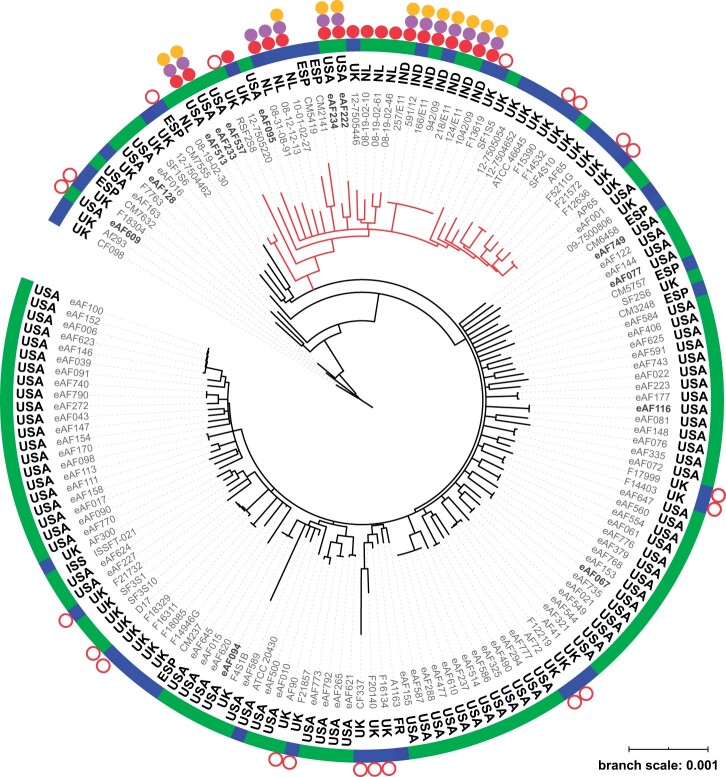
Neighbor-joining tree of environmental and clinical isolates of *Aspergillus fumigatus.* Whole-genome sequences from Georgia and Florida agricultural sites (this study, eAFXXX), were analyzed along with publicly available data (Supplementary Table S3). Af293 was used as the reference genome. Country of origin is listed next to each isolate (ESP, Spain; FR, France; IND, India; ISS, International Space Station; NL, Netherlands; UK, United Kingdom; USA, United States). Only branches with 100% bootstrap support based on 100 replicates are shown. Green bars indicate environmental isolates. Blue bars indicate clinical isolates. Solid red circles indicate pan-azole-resistant isolates with *cyp51A* TR mutations. Open red circles indicate azole-resistant isolates without TR mutations. Orange circles indicate isolates with *cytB* G143A mutation conferring resistance to QoI fungicides. Violet circles indicate *benA* F219Y mutation conferring resistance to MBC fungicides. Red branches indicate well-supported (100% bootstrap) pan-azole-resistant clade. Isolates with names shown in bold were assayed for growth on QoI- and MBC-amended media.

To better understand the relationship of Georgia and Florida agricultural isolates to worldwide environmental and clinical isolates, we performed whole genome sequencing on 89 isolates representing all of our field sites and combined them with 69 publicly available whole genome sequences to construct a neighbor-joining tree (Figure 1; Supplementary Table S3). Clinical and environmental azole-resistant isolates (Figure 1, open or closed red circles) from the United States (USA), United Kingdom (UK), Spain (ESP), the Netherlands (NL), and India (IND) were distributed throughout the tree; however, the four pan-azole-resistant isolates from this study that carried *cyp51A* TR alleles (Figure 1, closed red circles) clustered into a well-supported (100% bootstrap) clade with clinical and environmental isolates carrying *cyp51A* TR alleles from the UK, NL, and IND (Figure 1, red branches). This clade also included azole-sensitive isolates from diverse geographic origins. Although *A. fumigatus* does not show population structure by geographic or environmental origin, our data support population structure by pan-azole resistance. The genetic relatedness of pan-azole-resistant isolates across geographic locations and environment types has been previously described (Snelders *et al.* 2009; Abdolrasouli *et al.* 2015; Sewell *et al.* 2019; Etienne *et al.* 2021; Rhodes *et al.* 2021) and suggests that there is a barrier to gene flow or some other genetic predisposition in this pan-azole-resistant clade allowing *cyp51A* TR mutations to arise and/or persist.

We reasoned that if isolates of *A. fumigatus* in patients had evolved azole-resistance in agricultural settings, they might also have acquired resistance to other classes of fungicides. To determine if azole-resistant isolates carried alleles conferring resistance to QoI and MBC fungicides, we searched the genomes of our agricultural isolates along with the genomes of publicly available *A. fumigatus* isolates (Supplementary Table S3). We detected the *cytB* G143A allele known to cause QoI resistance (Figure 1, orange circles and Table 2) and/or the *benA* F219Y allele known to cause MBC resistance (Figure 1, violet circles and Table 2) in 20 clinical and environmental isolates, including four from Georgia agricultural sites. Only pan-azole-resistant isolates with the *cyp51A* TR allele also had *cytB* G143A and *benA* F219Y alleles. To verify that these alleles were associated with fungicide resistance, we tested growth on media amended with QoI or MBC fungicides for the four Georgia isolates carrying *cytB* and *benA* resistance alleles (eAF222, eAF233, eAF234, and eAF513) and nine Georgia isolates carrying fungicide-sensitive alleles (eAF67, eAF77, eAF94, eAF95, eAF116, eAF128, eAF537, eAF609, and eAF749). The isolates that were assayed for QoI and MBC resistance were genetically different (Figure 1, isolate names in bold) and came from both the clade with the pan-azole-resistant isolates (Figure 1, red branches) and the clade that did not include these isolates. Only isolates with the *cytB* G143A mutation grew on medium with the QoI fungicide azoxystrobin and only isolates with the *benA* F219Y mutation grew on medium with the MBC fungicide benomyl (Supplementary Figure S2 and Table S4).

**Table 2:** Mutations associated with fungicide resistance in pan-azole-resistant *A. fumigatus*

Isolate	Source	*cyp51A—*azoles^a^	*cytB—*QoI^b^	*benA—*MBC^c^
**A1163**	Clinic	WT	WT	WT
**eAF222**	Environment, 2018 USA	TR_46_/Y121F/T289A	G143A	F219Y
**eAF233**	Environment, 2018 USA	TR_46_/Y121F/T289A	G143A	F219Y
**eAF234**	Environment, 2018 USA	TR_46_/Y121F/T289A	G143A	F219Y
**eAF513**	Environment, 2018 USA	TR_46_/Y121F/T289A	G143A	F219Y
**08-12-12-13**	Clinic, 2003 Netherlands	TR_34_/L98H/	WT	F219Y
**08-31-08-91**	Clinic, 2004 Netherlands	TR_34_/L98H	WT	F219Y
**08-36-03-25**	Clinic, 2005 Netherlands	TR_34_/L98H	WT	F219Y
**10-01-02-27**	Clinic, 2010 Netherlands	TR_34_/L98H	G143A	F219Y
**Afu 942/09**	Clinic, 2009 India	TR_34_/L98H	G143A	F219Y
**Afu 1042/09**	Clinic, 2009 India	TR_34_/L98H	G143A	F219Y
**Afu 124/E11**	Environment, 2011 India	TR_34_/L98H	G143A	F219Y
**Afu 166/E11**	Environment, 2011 India	TR_34_/L98H	G143A	F219Y
**Afu 218/E11**	Environment, 2011 India	TR_34_/L98H	G143A	F219Y
**Afu 257/E11**	Environment, 2011 India	TR_34_/L98H	G143A	F219Y
**Afu 343/P11**	Clinic, 2011 India	TR_34_/L98H	G143A	F219Y
**Afu 591/12**	Clinic, 2012 India	TR_34_/L98H	G143A	F219Y
**DI 15-96**	Clinic, 2008 USA	TR_46_/Y121F/T289A	G143A	F219Y
**DI 15-102**	Clinic, 2010 USA	TR_34_/L98H	WT	F219Y
**DI 15-106**	Clinic, 2012 USA	TR_46_/Y121F/T289A	G143A	F219Y
**DI 15-116**	Clinic, 2014 USA	TR_34_/L98H	WT	F219Y

a GenBank accession number EDP50065 from azole-sensitive isolate A1163 was used as wildtype for Cyp51A. Isolates 08-12-12-13 and 08-36-03-25 also carried S297T/F495I mutations for Cyp51A, but these have not been associated with azole resistance.

b GenBank accession number YP_005353050 from azole-sensitive isolate A1163 was used as wildtype for CytB. All *cyp51A* TR mutants also carried V13I/I119V mutations for CytB, but these have not been associated with QoI resistance.

c GenBank accession number EDP56324 from azole-sensitive isolate A1163 was used as wildtype for BenA.

Twenty of the 25 pan-azole-resistant isolates included in our study were multifungicide resistant and carried alleles for QoI resistance, MBC resistance, or both (Table 2 and Figure 1). We analyzed six clinical isolates for fungicide-resistance alleles (Table 2 and Supplementary Table S3, italicized isolates), but they were not included in the phylogenetic analysis (Figure 1) because there was a substantial amount of missing data in the WGS sequence alignments. All QoI-resistant and MBC-resistant isolates were also pan-azole-resistant with TR alleles in *cyp51A* and clustered in the well-supported pan-azole clade (Figure 1, red branches, 100% bootstrap support). Eight of these multifungicide-resistant isolates were from agricultural settings in the USA and India and 12 were from clinical settings in the USA, The Netherlands, and India. Seven of these clinical isolates carried alleles conferring resistance to both MBC and QoI fungicides (Table 2). Of particular note, the three clinical and four environmental multifungicide-resistant isolates from India included in our phylogenetic analysis are nearly genetically identical (Figure 1).

## Discussion

We have shown that 86% of the pan-azole-resistant *A. fumigatus* isolates from patients in different countries are multifungicide-resistant and carry the allele for resistance to MBC fungicides and five of those also carry the allele for resistance to QoI fungicides used exclusively for plant protection. This shows that these isolates came from an agricultural environment and is additional evidence for an agricultural origin of at least some clinical pan-azole-resistant isolates. The *cytB* G143A and *benA* F219Y alleles have been shown to underlie resistance to QoI and MBC fungicides, respectively, for numerous fungal species (Gisi *et al.* 2002; Fisher and Meunier 2005; Fernandez-Ortuno *et al.* 2008; Fontaine *et al.* 2009; Lucas *et al.* 2015; Hawkins and Fraaije 2016; Pieczul and Wąsowska 2017; Forcelini *et al.* 2018) including for *A. fumigatus* in work published while this study has been under review (Fraaije *et al.* 2020; Gonzalez-Jimenez *et al.* 2021). These studies from other groups further support our finding that these alleles confer resistance to the corresponding fungicides.

It should be noted that not all pan-azole-resistant isolates in our study showed this signature of agricultural origin. Two of 14 (Figure 1 and Table 2) pan-azole-resistant clinical isolates did not carry MBC or QoI fungicide-resistance alleles raising the possibility that they could have originated from nonagricultural environments. However, since 3 of 11 pan-azole-resistant isolates from agricultural settings also lacked MBC or QoI alleles, it is also possible that clinical azole resistance might have originated in agricultural settings that did not have high levels of these specific fungicides.

We have shown that multifungicide-resistant *A. fumigatus* isolates are widely distributed across the globe (USA, NL, and IND) in both the environment and the clinic. This finding is supported by a recently published paper from another group (Gonzalez-Jimenez *et al.* 2021). We also found resistance to MBC and QoI fungicides exclusively in a single clade and always in combination with pan-azole resistance. It has been suggested that the genetic structure in populations of *A. fumigatus* could be driven by fungicide resistance (Rhodes *et al.* 2021). Beyond strongly supporting the agricultural origin of clinical pan-azole resistance in *A. fumigatus*, our results suggest that the unique genetics of the pan-azole clade enable the evolution and/or persistence of antimicrobial resistance mutations.

Recently, Fraaije *et al.* (2020) have shown that multifungicide-resistant isolates appear to be fit in agricultural environments where multiple fungicide classes are used. Asexual reproduction in the *A. fumigatus* pan-azole clade appears to allow multiple fungicide-resistance alleles to remain linked in the genome reminiscent of the accumulation of multidrug resistance mutations in human-pathogenic bacteria in agricultural environments (Klemm *et al.* 2018; McMillan *et al.* 2019). And, as seen in multidrug-resistant bacteria, multifungicide-resistant isolates may continue to accumulate resistance alleles as new single-site mode of action antimicrobials are introduced. Indeed, there is already evidence that multifungicide-resistant *A. fumigatus* isolates from the environment have acquired resistance to one of the newest classes of fungicides, the succinate dehydrogenase inhibitors (Fraaije *et al.* 2020). The linkage of resistance alleles raises the alarming possibility that the use of MBCs, QoIs, or other new single-site mode of action fungicides against plant-pathogenic fungi could increase the abundance of pan-azole-resistant *A. fumigatus* in the environment and lead to more multifungicide-resistant isolates in the clinic. The emergence of multifungicide-resistant *A. fumigatus* isolates severely limits the usefulness of single-site fungicides to manage plant-pathogenic fungi while still preserving the clinical usefulness of azoles. Effective fungicides with low environmental toxicity that do not lead to the rapid development of resistance are urgently needed for use in agricultural settings in order to preserve the usefulness of azoles against *A. fumigatus* in the clinic.

## Data availability

All environmental *Aspergillus fumigatus* sequence files (eAF) underlying this article are available in the NCBI Biosample database (https://www.ncbi.nlm.nih.gov/biosample/) [accession number(s) SAMN19975862–SAMN19975957]. All other sequences are publicly available at the sources noted in *Materials and*  *methods* and the legends to the corresponding figures.

Supplementary material is available at *G3* online.

## Supplementary Material

jkab427_Supplementary_Material

jkab427_Supplementary_Figure_Legends
