# Supplementary material for: Evidence for the agricultural origin of resistance to multiple antimicrobials in Aspergillus fumigatus, a fungal pathogen of humans
Source: G3 (Bethesda). 2021 Dec 13;12(2):jkab427. doi: 10.1093/g3journal/jkab427 (PMC9210323; doi:10.1093/g3journal/jkab427)
Supplement: jkab427_Supplementary_Material [file jkab427_supplementary_material.pdf]

Supplementary Figures for

Evidence for the agricultural origin of resistance to multiple antimicrobials in *Aspergillus fumigatus*, a fungal pathogen of humans

S. Earl Kang[1, †], Leilani G. Sumabat[2, §], Tina Melie[2, ‡], Brandon Mangum[1,2], Michelle Momany[1, *], Marin T. Brewer[2, *]

Figures S1-S2
Tables S1-S4

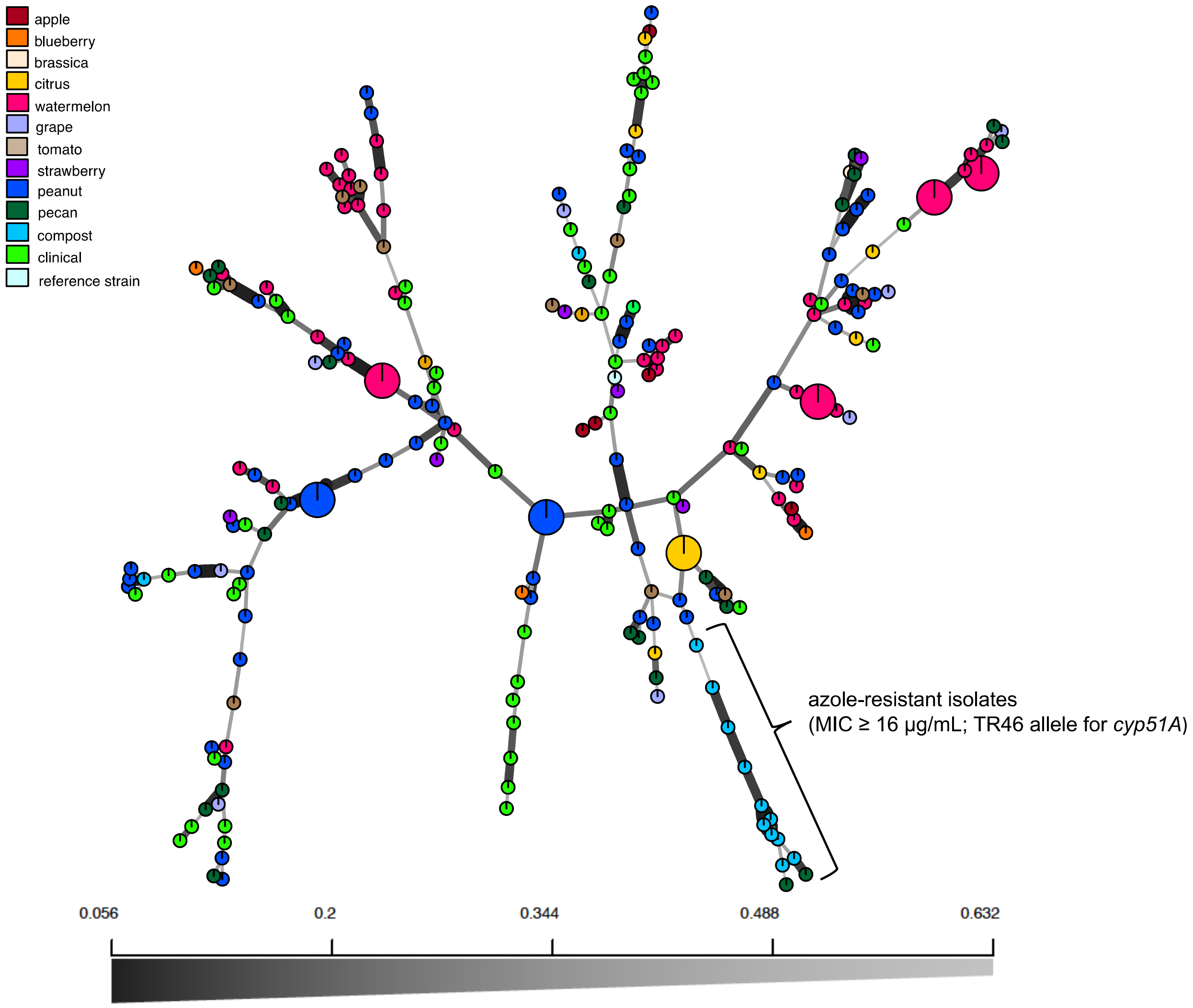

Figure S1. Minimum spanning network based on Bruvo's genetic distance of agricultural and clinical isolates of *A. fumigatus* from Georgia and Florida. Isolates (168 agricultural and 48 clinical) were genotyped with 9 STRAf markers. Each circle represents a unique haplotype and the size of the circle represents the relative frequency of detection. The color of each circle represents the environment where the isolate was collected. Thicker lines represent shorter genetic distances. Individuals with the TR46/Y121F/T289A allele for cyp51A are shown in the lower right.

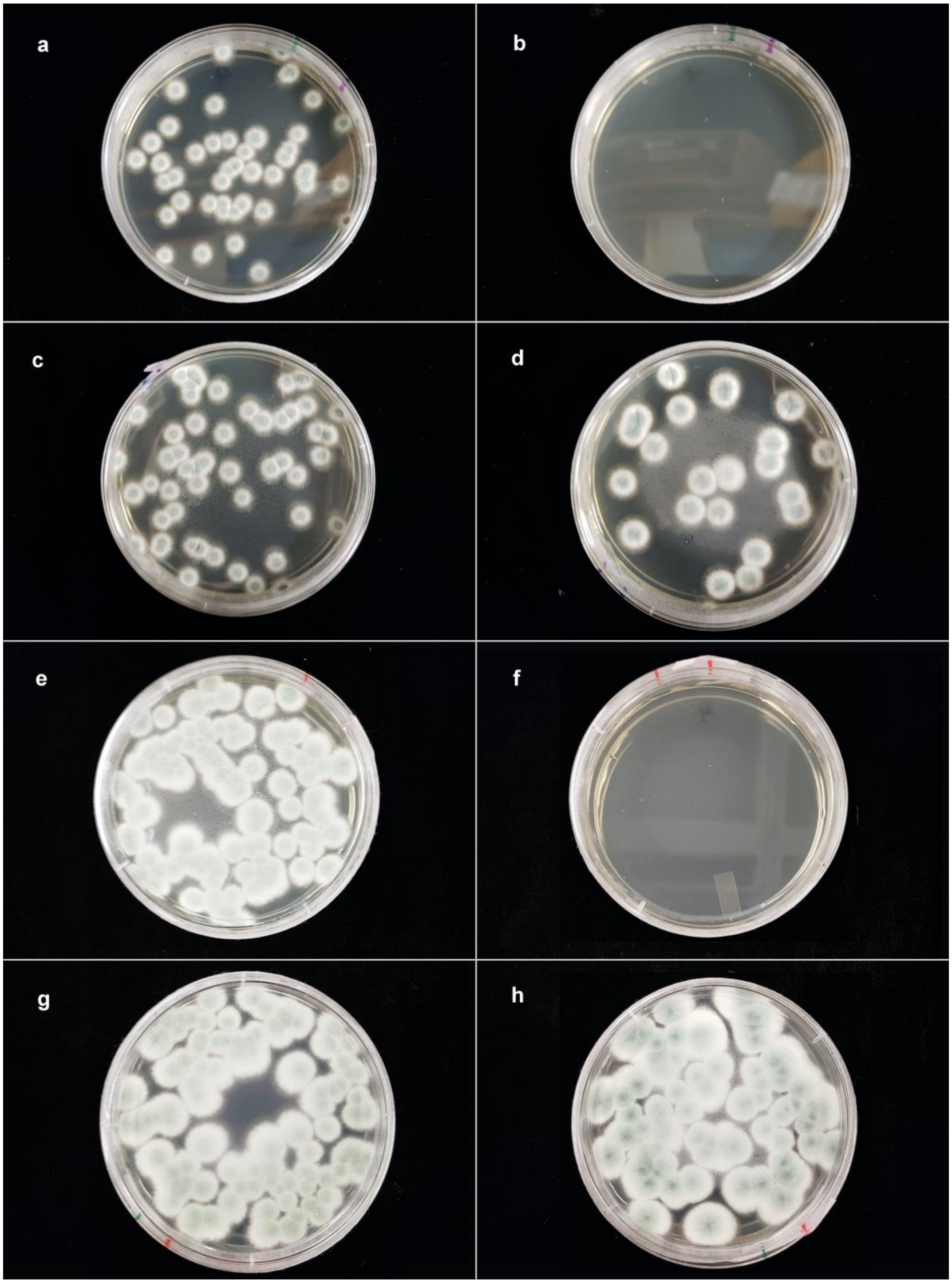

Figure S2. Pan-azole-resistant *A. fumigatus* (cyp51A TR46/Y121F/T289A) with cytB G143A and benA F219Y mutations are resistant to quinone outside inhibitor (QoI) and benzimidazole (MBC) fungicides. Left column (a, c, e, g) multi-fungicide-resistant isolate eAF222. Right column (b, d, f, h) sensitive isolate eAF94. (a, b) SDA (Sabouraud dextrose agar) + the QoI fungicide azoxystrobin + salicylhydroxamic acid (SHAM). (c, d) SDA medium + SHAM. (e,f) SDA medium + the MBC fungicide benomyl. (g, h) SDA medium.

Table S1. Sampling of *A. fumigatus* strains from agricultural sites.

Crop and/or substrate sampled[a]	Location(s) sampled	Sampling date(s) MM/DD/YY (no. sites[b])	Isolates collected, no. grew on TEB-amended plates	MIC[c]		WGS strains[d] (*azole-resistant)	No. isolates genotyped at *cyp51A* & STR*Af*[e]
				Sensitive	Resistant (MIC values)		
Apple (soil & plant debris)	Union Co., GA	08/21/17 (1)	9, 4	5	0	eAF76, eAF77	5
Blueberry (soil & plant debris)	Clinch Co., GA<br>Bacon Co., GA	01/17/18 (3)<br>01/18/18 (2)	1, 0<br>16, 0	1<br>2	0<br>0	eAF584	0<br>1
Citrus (soil & plant debris)	Polk Co., FL	03/28/18 (13)	64, 8	11	0	eAF735, eAF743, eAF749, eAF768, eAF770, eAF773, eAF776, eAF777, eAF790, eAF792	10
Watermelon (soil & plant debris)	Crisp Co., GA<br>Hillsborough Co., FL	06/07/17 (1)<br>03/27/18 (1)	3, 0<br>2, 2	1<br>1	0<br>0	eAF1<br>eAF740	1<br>1
Watermelon (soil & plant debris)	Crisp Co., GA	07/07/17 (1)<br>08/01/17 (1)<br>08/27/17 (1)	0, 0<br>18, 7<br>34, 24	0<br>9<br>25	0<br>0<br>0	eAF6, eAF10, eAF15, eAF16, eAF17, eAF39, eAF43	0<br>9<br>25
Compost pile	Clarke Co., GA	12/06/17 (1)	22, 12	2	11 (1 TEB & VOR = 16 μg/mL; 10 TEB & VOR > 16 μg/mL)	eAF222*, eAF223, eAF227, eAF233*, eAF234*	13
Grape (soil & plant debris)	Union Co., GA	08/21/17 (1)	14, 7	9	0	eAF21, eAF22, eAF61, eAF67, eAF72	9
Tomato (soil & plant debris)	Hillsborough Co., FL	03/27/18 (3)	35, 3	7	0	eAF620, eAF621, eAF623, eAF624, eAF625, eAF645, eAF647	7
Strawberry (soil & plant debris)	Hillsborough Co., FL	03/27/18 (1)	25, 3	5	1 (TEB = 4 μg/mL)	eAF586, eAF587, eAF589, eAF591, eAF609*, eAF610	6
Peanut (soil & plant debris)	Tift Co., GA	10/20/17 (6)	28, 18	22	0	eAF90, eAF91, eAF94, eAF95, eAF111, eAF113 eAF144, eAF148, eAF152, eAF153, eAF154,	22

Peanut (plant debris)	Tift Co., GA	10/20/17 (4)	59, 27	33	0	eAF81, eAF98, eAF100, eAF105, eAF116, eAF122, eAF128, eAF146, eAF147, eAF155, eAF158, eAF163, eAF177, eAF170	32
	Turner Co., GA	10/20/17 (1)	4, 2	3	0		3
Pecan (plant debris; two sites were processing facilities)	Dougherty Co., GA	12/13/17 (11)	294, 6	19	1 (TEB & VOR MIC > 16 µg/mL)	eAF263, eAF265, eAF272, eAF288, eAF321, eAF325, eAF365, eAF379, eAF406, eAF477, eAF490, eAF500, eAF513*, eAF514, eAF537, eAF544, eAF549, eAF554, eAF560	19
Pecan (soil & plant debris)	Dougherty Co., GA	12/13/17 (2)	28, 0	2	0	eAF294, eAF335	2
Organic cucurbits (soil & plant debris)	Oconee Co., GA	12/06/17 (1)	28, 0	1	0		0
Organic brassicas (soil & plant debris)	Clarke Co., GA	12/06/17 (1)	16, 0	1	0	eAF237	1
Total		56 sites	700, 123	159	13	92	166

[a]Four samples were collected from distinct locations at each site; soil & plant debris indicates that soil was sampled and decaying plant debris on the soil surface was included in the sample.

[b]Each site was defined as a different field location, different crop at the same field location, or different triazole fungicide treatment

[c]All strains that grew on the TEB-amended medium were assayed for MIC. Additionally, 1 or 2 strains per site that did not grow on the amended medium were assayed for MIC for comparison. Isolates were considered resistant if TEB, ITC or VOR MIC values were > 2 µg/mL, or POS > 1 µg/mL; otherwise they were considered sensitive. MIC assays were conducted twice for all resistant isolates and most sensitive isolates. Most often, there was no variation between replicates, but if there was a difference, the greater MIC is presented here.

[d]For whole genome sequencing (WGS) and phylogenetic analyses, 1 strain that grew and 1 strain that did not grow on TEB-amended medium that had been assayed for MIC were analyzed per site. WGS from three isolates (eAF105, eAF263, eAF365) were not included in the NJ tree due to excessive missing data.

[e]*cyp51A* genotypes were analyzed from all WGS strains and *cyp51A* genotypes for all remaining strains that grew on TEB-amended medium were analyzed by PCR and Sanger sequencing. All strains were genotyped with STR*Af* microsatellite markers.

Table S2. Minimum inhibitory concentrations (MIC)[a] for *A. fumigatus* (*n* = 172) isolated from agricultural environments in Georgia and Florida where azole fungicides were applied.

Azole	Final Drug Concentration (µg/mL)								
	**>16**	**16**	**8**	**4**	**2**	**1**	**0.5**	**0.25**	**<0.25**
**Tebuconazole**	11	1		1	85	68	6		
**Itraconazole**					11	140	21		
**Voriconazole**	12					1	81	72	6
**Posaconazole**						15	93	58	6

[a]Isolates were assayed for MIC once (some sensitive isolates) or twice (all resistant and some sensitive isolates). Most often, there was no variation between replicates, but if there was a difference, the greater MIC is presented here.

Table S3. Publicly available genome sequence data used in this study for neighbor-joining tree and mining for antifungal-resistance genes and mutations (italics).

**NCBI_SRA_ ID**	**Taxa_ID**	**Sampling Location**	**Environ (E) /Clinical (C)**	**Azole-Res (R) /Sens(S)**	**Reference**
ERR769506	08-12-12-13	Netherlands	C	R	doi: 10.1128/mBio.00536-15
ERS663179	08-19-02-10	Netherlands	E	R	doi: 10.1128/mBio.00536-15
ERS663176	08-19-02-30	Netherlands	E	S	doi: 10.1128/mBio.00536-15
ERR769512	08-19-02-46	Netherlands	E	R	doi: 10.1128/mBio.00536-15
ERR769509	08-19-02-61	Netherlands	E	R	doi: 10.1128/mBio.00536-15
ERR769508	08-31-08-91	Netherlands	C	R	doi: 10.1128/mBio.00536-15
ERS663166	09-7500806	UK	C	S	doi: 10.1128/mBio.00536-15
ERR769511	10-01-02-27	Netherlands	C	R	doi: 10.1128/mBio.00536-15
ERS663168	12-7504462	UK	C	S	doi: 10.1128/mBio.00536-15
ERS663167	12-7504652	UK	C	S	doi: 10.1128/mBio.00536-15
ERS663169	12-7505054	UK	C	S	doi: 10.1128/mBio.00536-15
ERS663165	12-7505220	UK	C	R	doi: 10.1128/mBio.00536-15
ERS663164	12-7505446	UK	C	R	doi: 10.1128/mBio.00536-15
ERR232426	A1163	France	C	S	NCBI SRA
ERS663170	Af293	UK	C	S	doi: 10.1128/mBio.00536-15
ERX207014	AF300	UK	C	S	NCBI SRA
SRS375791	AF41	UK	C	S	NCBI SRA
ERS663171	AF65	UK	C	S	doi: 10.1128/mBio.00536-15
SRR617721	AF72	UK	C	R	doi: 10.1128/AAC.41.6.1364
SRR617722	AF90	UK	E	R	doi: 10.1128/AAC.41.6.1364
ERS663181	Afu_1042/09	India	C	R	doi: 10.1128/mBio.00536-15
ERS663184	Afu_124/E11	India	E	R	doi: 10.1128/mBio.00536-15
ERS663185	Afu_166/E11	India	E	R	doi: 10.1128/mBio.00536-15
ERS663187	Afu_218/E11	India	E	R	doi: 10.1128/mBio.00536-15
ERS663186	Afu_257/E11	India	E	R	doi: 10.1128/mBio.00536-15

ERS663183	Afu_591/12	India	C	R	doi: 10.1128/mBio.00536-15
ERS663180	Afu_942/09	India	C	R	doi: 10.1128/mBio.00536-15
ERS216929	AP65	UK	E	S	NCBI SRA
SRR7418943	ATCC_204305	USA	C	S	doi: 10.3390/genes9070363
SRR7418935	ATCC_46645	UK	C	S	doi: 10.3390/genes9070363
ERS216946	CF098	UK	C	S	NCBI SRA
ERR232405	CF337	UK	C	R	NCBI SRA
SRR7418947	CM2141	Spain	C	S	doi: 10.3390/genes9070363
SRR7418942	CM237	Spain	C	S	doi: 10.3390/genes9070363
SRR7418945	CM3248	Spain	C	S	doi: 10.3390/genes9070363
SRR7418944	CM5419	Spain	C	S	doi: 10.3390/genes9070363
SRR7418949	CM5757	Spain	C	S	doi: 10.3390/genes9070363
SRR7418936	CM6458	Spain	C	S	doi: 10.3390/genes9070363
SRR7418938	CM7555	Spain	C	R	doi: 10.3390/genes9070363
SRR7418941	CM7632	Spain	C	S	doi: 10.3390/genes9070363
ERR232430	D17	UK	E	R	NCBI SRA
SRR617724	F12219	UK	C	R	doi: 10.3201/eid1507.090043
SRR617725	F12636	UK	C	R	doi: 10.3201/eid1507.090043
SRR617727	F13619	UK	C	R	doi: 10.3201/eid1507.090043
SRR617729	F14403	UK	C	R	doi: 10.3201/eid1507.090043
SRR617731	F14532	UK	C	R	doi: 10.3201/eid1507.090043
SRS375803	F14946G	UK	C	S	doi: 10.3201/eid1507.090043
SRR617733	F15390	UK	C	R	doi: 10.3201/eid1507.090043
SRR617735	F16134	UK	C	R	doi: 10.3201/eid1507.090043
SRS375808	F16311	UK	C	S	doi: 10.3201/eid1507.090043
ERR232417	F17999	UK	C	R	doi: 10.3201/eid1507.090043
SRS375813	F18085	UK	C	S	doi: 10.3201/eid1507.090043
ERR232439	F18304	UK	C	R	doi: 10.3201/eid1507.090043
ERR232418	F18329	UK	C	R	doi: 10.3201/eid1507.090043
ERR232413	F20140	UK	C	R	doi: 10.3201/eid1507.090043
ERR232440	F21572	UK	C	R	doi: 10.3201/eid1507.090043
ERR232414	F21732	UK	C	R	doi: 10.3201/eid1507.090043

ERR232421	F21857	UK	C	R	doi: 10.3201/eid1507.090043
ERS216942	F4S1B	UK	E	S	NCBI SRA
SRS375812	F5211G	UK	C	S	doi: 10.3201/eid1507.090043
SRS375815	F7763	UK	C	S	doi: 10.3201/eid1507.090043
SRR4002443	ISSFT-021	International Space Station	E	S	doi: 10.1128/mSphere.00227-16
ERR232441	RSF2S8	UK	E	R	NCBI SRA
ERS216922	SF1S5	UK	E	S	NCBI SRA
ERS216948	SF1S6	UK	E	S	NCBI SRA
ERS216938	SF2S6	UK	E	S	NCBI SRA
ERS216937	SF3S1	UK	E	S	NCBI SRA
ERS216930	SF3S10	UK	E	S	NCBI SRA
ERS216918	SF4S10	UK	E	S	NCBI SRA
ERR769507	*08-36-03-25*	*Netherlands*	*C*	*R*	*doi: 10.1128/mBio.00536-15*
ERS663182	*Afu_343/P/11*	*India*	*C*	*R*	*doi: 10.1128/mBio.00536-15*
SRR7841978	*DI_15-102*	*USA*	*C*	*R*	*doi: 10.1128/mBio.00437-20*
SRR7841983	*DI_15-106*	*USA*	*C*	*R*	*doi: 10.1128/mBio.00437-23*
SRR7841993	*DI_15-96*	*USA*	*C*	*R*	*doi: 10.1128/mBio.00437-36*
SRR7842000	*DI_15-116*	*USA*	*C*	*R*	*doi: 10.1128/mBio.00437-31*

Table S4. Fungicide resistance genotypes and growth phenotypes for agricultural isolates from Georgia and Florida.

Isolate	*cyp51A* genotype	Growth on azole[a]	*cytB* genotype	Growth on QoI[b]	*benA* genotype	Growth on MBC[c]
eAF67	WT	-	WT	-	WT	-
eAF77	WT	-	WT	-	WT	-
eAF94	WT	-	WT	-	WT	-
eAF95	WT	-	WT	-	WT	-
eAF116	WT	-	WT	-	WT	-
eAF537	WT	-	WT	-	WT	-
eAF749	WT	-	WT	-	WT	-
eAF128	Y46F/V172M/T248N/ E255D/K427E	-	WT	-	WT	-
eAF609	Y46F/V172M/T248N/ E255D/K427E	-	WT	-	WT	-
eAF222	TR_{46}/Y121F/T289A	+	G143A	+	F219Y	+
eAF233	TR_{46}/Y121F/T289A	+	G143A	+	F219Y	+
eAF234	TR_{46}/Y121F/T289A	+	G143A	+	F219Y	+
eAF513	TR_{46}/Y121F/T289A	+	G143A	+	F219Y	+

[a] One hundred conidia were inoculated to solid medium containing 3 µg/ml tebuconazole (TEB) and incubated at 37°C for 23-24 h. Also, isolates with '+' had MICs $\geq$16 µg/ml and isolates with '-' had MICs $\leq$4 µg/ml in TEB liquid medium (Table 1).
[b] One hundred conidia were inoculated to solid medium containing 10 µg/ml azoxystobin and incubated at 37°C for 23-24 h.
[c] One hundred conidia were inoculated to solid medium containing 10 µg/ml benomyl and incubated at 37°C for 23-24 h.
