# Supplementary material for: Evidence for the agricultural origin of resistance to multiple antimicrobials in Aspergillus fumigatus, a fungal pathogen of humans
Source: G3 (Bethesda). 2021 Dec 13;12(2):jkab427. doi: 10.1093/g3journal/jkab427 (PMC9210323; doi:10.1093/g3journal/jkab427)
Supplement: jkab427_Supplementary_Figure_Legends [file jkab427_supplementary_figure_legends.docx]

**Figure S1. Minimum spanning network based on Bruvo’s genetic distance of agricultural and clinical isolates of *A. fumigatus* from Georgia and Florida.** Isolates (168 agricultural and 48 clinical) were genotyped with 9 STRAf markers. Each circle represents a unique haplotype and the size of the circle represents the relative frequency of detection. The color of each circle represents the environment where the isolate was collected. Thicker lines represent shorter genetic distances. Individuals with the TR46/Y121F/T289A allele for cyp51A are shown in the lower right.

**Figure S2.** Pan-azole-resistant *A. fumigatus* (cyp51A TR46/Y121F/T289A) with cytB G143A and benA F219Y mutations are resistant to quinone outside inhibitor (QoI) and benzimidazole (MBC) fungicides. Left column (a, c, e, g) multi-fungicide-resistant isolate eAF222. Right column (b, d, f, h) sensitive isolate eAF94. (a, b) SDA (Sabouraud dextrose agar) + the QoI fungicide azoxystrobin + salicylhydroxamic acid (SHAM). (c, d) SDA medium + SHAM. (e,f) SDA medium + the MBC fungicide benomyl. (g, h) SDA medium.
